# A Rodent Model of Mild Neonatal Hypoxic Ischemic Encephalopathy

**DOI:** 10.3389/fneur.2021.637947

**Published:** 2021-05-05

**Authors:** Julien Gotchac, Laura Cardoit, Muriel Thoby-Brisson, Olivier Brissaud

**Affiliations:** ^1^Institut des Neurosciences Cognitives et Intégratives d'Aquitaine, CNRS UMR 5287, University of Bordeaux, Bordeaux, France; ^2^Pediatric Intensive Care Unit, Teacher's hospital of Children Pellegrin, Bordeaux, France

**Keywords:** hypoxic ischemic encephalopathy, cardiac arrest, cardiopulmonary resuscitation, development, brain injury

## Abstract

In the brain of full-term newborns, Hypoxic Ischemic Encephalopathy (HIE), a consequence of severe hypoxia and ischemia due to low cardiac output, is frequently observed and results in cerebral injuries with dramatic consequences for life. To investigate the physiopathology of HIE, several animal models have been developed, but none closely replicate human cases, mostly because they are based on a single carotid ligation protocol. In the present study we aimed to develop a novel and more accurate HIE model in juvenile (post-natal days (PND) 14–16) rats. For this, we induced a 9 min hypoxic cardiac arrest (CA) by stopping mechanical ventilation of intubated, ventilated and curarized rats followed by a cardiopulmonary resuscitation. To evaluate the consequences of the CA we performed radiological (cerebral MRI), behavioral (Open Field, Elevated Plus Maze, Fear Conditioning), and histological (Cresyl Violet and Fluoro-Jade B) testing on treated animals. We found that rats in the CA group developed an anxiolytic-like behavioral profile in adulthood without any locomotor impairment, nor memory deficits. However, MRI investigation performed early after CA failed to reveal any change in apparent diffusion coefficient (ADC) in brain tissue (including the hippocampus, striatum, and thalamus), suggesting no massive anatomical lesion had occurred. In contrast, signs of neurodegeneration were found in the Dentate Gyrus and the CA1 region of the hippocampus at day 1 post-CA, suggesting that the anxiolytic-like phenotype observed in adulthood could be related to an abnormal degeneration of this brain region beginning immediately after CA. Thus, our model, despite not representing a severe condition of HIE, nonetheless constitutes a potential model for studying mild, yet persistent and region-specific cerebral injury resulting from an acute oxygen deprivation.

## Introduction

Hypoxic ischemic encephalopathy (HIE) of the full-term newborn remains a significant cause of mortality and morbidity, with an estimated incidence range from 1.3 to 1.7 per 1,000 live birth (1–4). It is the third leading cause of death in children under 5 years of age (3) and can induce neurological disabilities of varying severity (1). In the full-term newborn, HIE causes specific lesions in areas of high energy demand that can differ depending on the degree of cerebral maturation at the time of the insult (5). There are two main types of injury. First, in the context of a moderate decrease in cerebral blood flow, cerebral lesions are mainly cortico-subcortical, para-sagittal (vascular frontier areas) through an antero-posterior vascular redistribution (6, 7) with a milder clinical phenotype outcome, typically without profound motor deficits (8). Second, in case of a sudden, acute hypoxic event and profound hypotension, adaptive mechanisms fail to develop. In these conditions, the areas of high energy requirement, i.e., the basal ganglia, thalamus, hippocampus, and cortico-spinal tracts, are the most affected (5, 6).

Most HIE rodent models are inspired by that developed by Rice and Vannucci in 1981 (9), itself deriving from Levine's original preparation (10). It involves a unilateral ligation of the common carotid artery followed by hypoxia in 7 post-natal day (PND) old rats, which triggers selective neuronal death or cerebral infarction in the ipsilateral cortex, striatum and/or hippocampus, as well as white matter necrosis. Whereas, this model has been widely used and has led to a better understanding of HIE pathophysiology (11), many improvements have subsequently been proposed, such as a bilateral carotid ligation with or without hypoxia (12), models incorporating reperfusion or models with intra-peritoneal injection of lipopolysaccharide (LPS) to induce an inflammatory response (13). However, these protocols all have major limitations: the focal distribution of cerebral injuries recapitulates more a neonatal stroke injury than the deeper and widespread damage encountered in neonates with a HIE. In 2004, Fink (14) reported an original model of pediatric cardiac arrest, with an 8-min hypoxic cardiovascular arrest resulting in an overall impairment of cerebral blood flow. They found ischemic neuronal lesions in the CA1 region of the hippocampus and in the cortex, as well as persistent memory disorders evaluated by Morris Watermaze. However, this protocol was applied to juvenile P16–P18 rats, which therefore had more advanced brain development than a full-term human newborn (15).

In this context, the aim of our work was to develop an original and practical model that mimics HIE human physiopathology as accurately as possible. We have chosen to adapt the model developed by Fink but making it less invasive, with a prolonged hypoxia duration and applied to younger rats (PND 14–16). In addition, we further assessed our model with MRI analysis, which has not been previously employed.

## Methods

### Animals

All experiments were conducted under the approval of the Ethics Committee of the University of Bordeaux (referral number: APAFIS#18661-2019012517121887) and the European Union directive 2010/63/EU.

Our experiments used Sprague-Dawley rats (*n* = 59) that were housed with their mother until experimental use or weaning (PND 21). After weaning, they were housed in pairs of same sex, in standard rat cages and with standard nutrient enrichment. The environment was controlled in terms of temperature, hygrometry, and 12/12 h artificial light cycle.

### Experimental Cardiac Arrest Procedure

Mixed-gender PND 14–16 Sprague Dawley rats were anesthetized with 4% isoflurane in an induction chamber ventilated with a 3.5 L/min airflow, until unconsciousness was reached. They were then weighed and placed on an intubation stand (Kent Scientific, Torrington, USA), while the inhaled anesthesia was maintained (airflow 1 L/min, Isoflurane 2%). Rats were then intubated with a 20 Ga angiocatheter, connected to the ventilator SAR-830 A/P (CWE Inc., Phymep, France), set with the following parameters: tidal volume (Vt) 12 ml/kg, respiratory rate (RR) 50/min, insufflation time (Ti) 0.4 s, and fraction of inspired Oxygen (FiO_2_) 21%. Correct positioning of the endotracheal tube was verified by the observation of a symmetrical thoracic inflation. The animals were then placed on a heating plate controlled by a thermal controller (TC-100 Temperature Controller, CWE Inc., Phymep, France) connected to a rectal temperature probe, with a target temperature of 37°C. Cardiac electrical activity was monitored using three ECG electrodes placed on the limbs and connected to a PowerLab monitor. This monitor was also connected to the ventilator, the thermal controller and a computer to record the following parameters: electrocardiogram (ECG), heart rate (HR), respiratory rate (RR), and temperature. 0.1 mg (2–3 mg/kg) of Rocuronium was administered intravenously (lateral caudal vein) to achieve complete neuromuscular blockade and thus prevent any spontaneous respiratory movement.

In the experimental group, the following cardiac arrest (CA) protocol was applied: animals were exposed to isoflurane for 6 min. The anesthetic was then stopped to allow a 2 min washout period before turning off the ventilator (16). After 8 min of hypoxia, an injection of 5 mcg/kg epinephrine was performed using retro-orbital access (17, 18) in order to reverse cardiac arrest and facilitate resuscitation. The retro-orbital site was used because it was a relatively safe procedure, and standard intravenous injection usually made in the lateral vein was too difficult in such young animals, especially under cardiac arrest. Then, ventilation was resumed after the 9 min of hypoxia using the ventilator controller, with the following parameters: Vt 15 ml/kg, RR 70/min, Ti 0.285 s and FiO2 1. Immediately afterwards, chest compressions were performed at a rate of 200/min. Once per minute, resuscitation was briefly interrupted to observe whether normal sinus activity had resumed. After 10 min of resuscitation, animals were declared dead if there was still no sign of sinus activity, or if resuscitation was successful, the ventilator parameters were gradually changed until the initial parameters were reached, except for FiO2 which was maintained at 1. When effective respiratory movements were observed, the animals were weaned from mechanical ventilation and extubated. They then received a subcutaneous injection of 10 ml/kg saline with 5% glucose to prevent dehydration since they were unable to feed by themselves. After 1 h of observation, they were placed back in their cage with their mother.

Rats in the control group underwent the identical procedure, with the exception of the cardiac arrest stage: they were intubated, curarized, and anesthesia inhalation was continued until 6 min post-injection. A placebo retro-orbital injection (0.9% NaCl) was also administrated.

### MRI Analysis

MRI experiments were performed with a 7 T Brucker BioSpec system (Ettlingen, Germany), equipped with a volume resonator (75.4 mm inner diameter, active length 70 mm) for excitation, and a 4-element (2 × 2)-phased array surface reception coil. Rats were anesthetized with isoflurane (1.5–2% in air) and positioned with the brain at the center of the Nuclear Magnetic Resonance coil. Animal breathing was monitored during the scanning session (SA Instruments, Stony Brook, NY). A B0 map was drawn with a field of view (FOV) of 30 × 30 × 30 mm. Diffusion Weighted Images (DWI) sequences were acquired with a pulsed gradient spin echo technique, with the following parameters: 50 slices of 0.5 mm thickness, FOV = 25 × 25 mm, matrix = 128 × 128, TE = 24.5 ms, TR = 3,000 ms, six directions of space with b = 1,000 s/mm^2^; four acquisitions. The acquisition time was 336 s. Anatomical images were obtained using a T2-weighted RARE sequence with the following parameters: 50 slices of 0.5 mm thickness, FOV = 25 × 25 mm, matrix = 256 × 256, TE = 50 ms, TR = 7,818 ms, RARE factor = 8; four acquisitions. The acquisition time was 16 min and 40 s. For each of these sequences, a macroscopic evaluation of injuries was performed on all sections. For each animal, three slices of DWI were chosen, where the following structures were the largest and most visible: striatum, hippocampus and thalamus. Within each of these structures, we measured mean Apparent Coefficient Diffusion (ADC) values (mm^2^/s) on a 2.4 mm^2^ square grid. MRI was performed at day 1 after the experimental procedure described above to assess for early injuries (in T2 and DWI) and 7–10 days later to assess for long-term tissue damage on the T2 sequences.

### Histological Analysis

After MRI scanning sessions, the animals were anesthetized with a Ketamine/Xelazine solution and then perfused intracardially and exsanguinated with a peristaltic pump conveying 0.9% NaCl followed by 4% PFA diluted in PBS. After this fixation phase, animals were decapitated, and the brain removed and post-fixed in 4% PFA overnight. Thereafter, the brains were placed for 48 h in a 20% PBS-sucrose solution and then frozen in isopenthane at −43°C to be subsequently sliced (30 or 50 μm thick) using a cryostat (Leica CM 3000).

#### Cresyl Violet Staining (19)

Coronal slices of interest were placed on a slide, dehydrated in a bath series with increasing alcohol concentration and then rehydrated in a bath series with decreasing alcohol concentration. They were then placed in a solution of Cresyl Violet for 5 min and then re-dehydrated in the same way. Finally, the slide tissue was cleared by several successive xylene baths. The stained sections were observed and photographed with a Nikon SMZ18 stereomicroscope.

#### Fluoro-Jade Labeling and Quantification (20)

Coronal slices (50 μm thick) mounted on gelatin coated slides were first immersed in a solution containing 1% NaOH in 80% alcohol for 5 min. Then slides were successively placed in 70% alcohol for 2 min, distilled water for 2 min and a solution of 0.06% potassium permanganate for 10 min. Slides were then rinsed again in distilled water for 2 min. The staining solution was prepared from a 0.01% stock solution of Fluoro-Jade B (Histo-Chem Inc., Jefferson AR or VWR) with 10 mg of the powder diluted in 100 ml distilled water. This staining solution was prepared freshly for each experiment with 4 ml of the stock solution added to 96 ml of 0.1% acid vehicle. After 20 min exposure to the staining solution, slides were rinsed 3 times in distilled water for 1 min each and thereafter were placed on a slide warmer until they were fully dry. In a final step, slides were cleared by immersion in xylene baths and cover-slipped in a non-aqueous non-fluorescent plastic mounting media (CoverQuick, VWR) (Shumed and Hopkins 2000). Images were acquired with a Zeiss confocal microscope LSM900 and analyzed with the Fiji software. Fluorescence intensities were measured from seven slices obtained from 3 to 4 different animals in the control and the CA groups. Measurements were performed on each half slice from three regions of interest: one region outside the hippocampus where no specific labeling was observed, thus defining the background intensity, one region encompassing the Dentate gyrus (DG), and one encompassing the CA1 part of the hippocampus. The variation of fluorescence was calculated for DG and CA1 relative to background (% ΔF/F).

### Behavioral Experiments

#### Open Field (21)

Animals were placed individually for 10 min in a white, low-lit square, and gridded arena with wooden borders and a 102 × 102 cm PVC floor surface, disinfected between each tested rat. A black mark was previously drawn on the heads of the rats with a simple make-up pencil, so that they could be spotted by the video tracking system consisting of a camera placed above the arena and connected to a computer (outside the room), thereby allowing the monitoring of the animal's position. Data were recorded using the ViewPoint software, with integration periods of 60 s. For the analysis, we artificially divided the Open Field into two areas: a central square of 40 × 40 cm and a peripheral area around. Three main measurements were collected: the total distance traveled, the time spent and the distance traveled in each area, and the number of entries into the central area. The latter two parameters are good indicators of anxiety since the central square represents a stressful area for rats that have an aversion to large, open, unfamiliar, and bright environments (22).

#### Elevated Cross Maze (ECM) (23)

The rats were placed individually for 5 min in an ECM, 62 cm above the ground, with two arms enclosed by a 40 cm high wall, perpendicular to two open arms, and a transition square between these four arms. The maze was made with dark PVC, measured 116 × 116 cm, and was placed in the middle of a low-lit room. The video tracking system was identical to that used for the Open Field analysis. Data were recorded using ViewPoint software without an integration period. For the analysis, we artificially divided the maze into five areas: one for each arm and one for the central square. The main information collected was the ratio between the time spent in the open arms and the total time spent in all arms (open + closed), noted [OA/(OA+ClA)], which is inversely correlated with anxiety (24).

#### Fear Conditioning Test (25, 26)

These experiments were conducted in two stages on consecutive days in 8 identical conditioning chambers (40 × 35 × 30 cm) made of one transparent plastic side and three gray PVC sides. A grid floor (27 metal bars) above a sawdust tray could deliver mild foot electric shocks (0.4 mA AC for 1 s, randomly distributed in arrays of 8 among the 27 metal bars). Stimuli were delivered by a computerized interface (Poly software, Imetronic). Each conditioning chamber was equipped with a miniature (30 × 30 × 32 mm) black and white video camera (SK-2005, OptoVision, Toulouse, France), centered overhead. The camera monitored the entire chamber through a 2.45 mm wide angle lens. Lighting was provided by four LED bulbs through a frosted plastic screen. A set of four cameras was connected to a Quad-type multiplexer (Computar QSMX-II) which combined their four inputs. The resulting video signal was sampled online by a PC type microcomputer equipped with a Scion LG3 video capture card (Scion Corporation, Frederick, Maryland). The principle of the test is to induce Pavlovian associative learning between an environmental context (the illuminated conditioning box) and an aversive stimulus (the electric shock), then to test the persistence of this learning 24 h later. On the 1st day (conditioning phase), the rats were placed individually in a conditioning chamber for a period of 8 min, which was inaugurated by switching on the chamber light. Five electric shocks were delivered at 120, 180, 260, 300, and 400 s. The next day, each rat was returned to the same conditioning box as the day before, but for a longer period (10 min) and without any electrical shock administration. For each animal, we assessed the rate of behavioral “freezing” every minute during both experimental conditions, which was scored manually with a stopwatch. Freezing was defined as the complete absence of movement except that required for respiration, this innate response being a measurement of the fear level in rodents (25, 26).

### Statistical Analysis

The results are presented as means ± SE. For behavioral tests with a dynamic component (Open Field and Fear conditioning test), we used a repeated measures ANOVA. For the other analyses, we applied mean comparison tests. Non-parametric tests were used when the population size was too small, when values were not following a normal distribution, or when there was no homogeneity of variance between groups. Note that due to an insufficient number of male rats having undergone a control procedure, we had to use a heterogeneous control group related to the inclusion of four healthy rats out of six. All statistical analyses were performed with R software, with a probability threshold for rejection of the null hypothesis (*p*) of 0.05.

## Results

### Experimental Cardiac Arrest Procedure

**Figure 1:**
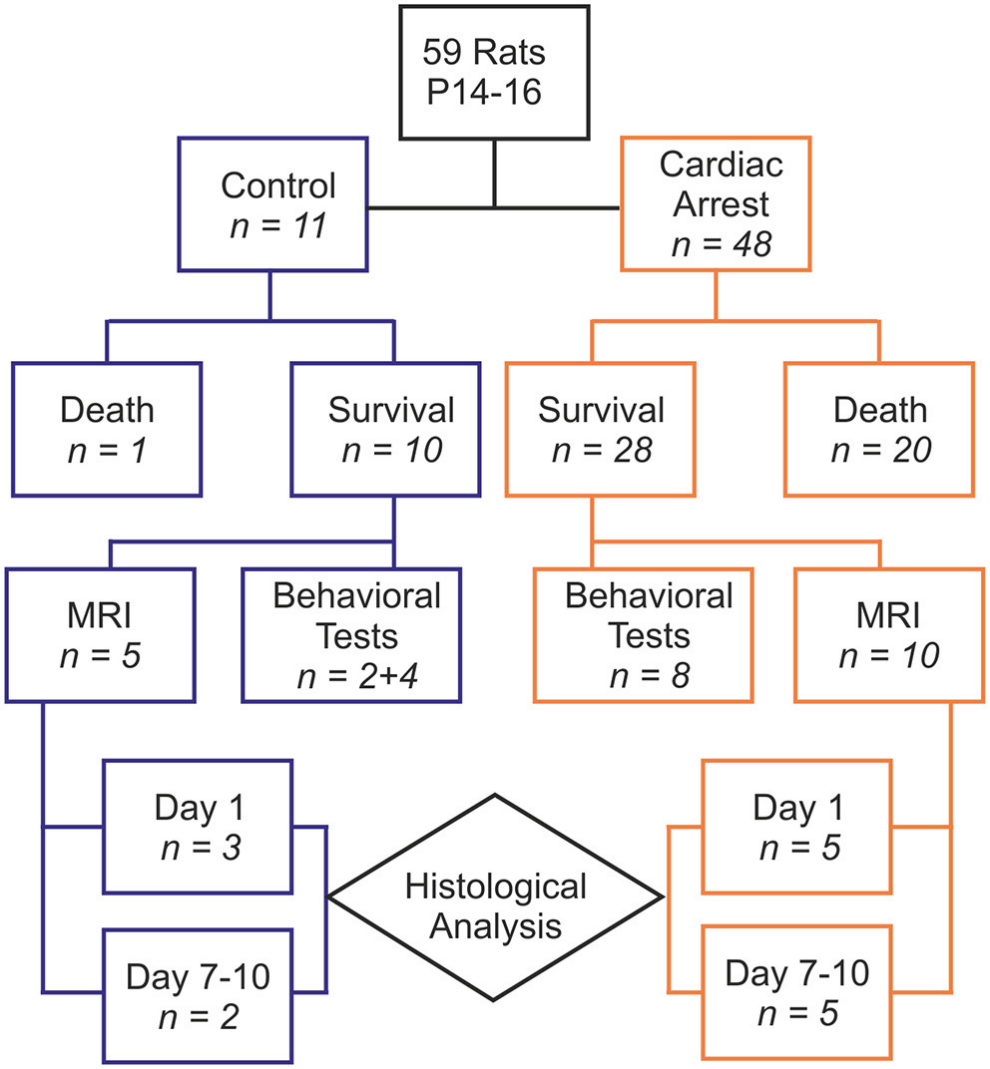
Diagram showing the experimental paradigm of the study with the group distributions and numbers of animals designated to each test performed.

Fifty-nine animals were included in our study, with 48 comprising the cardiac arrest (CA) group and 11 in the control group (6 rats that died from tracheal injury following intubation difficulties during the first procedures were not considered). The experimental flowchart for the entire study is presented in Figure 1. In the CA group, mortality was quite high (41.67%), due to resuscitation failure from persistent atrioventricular dissociation (*n* = 14) and deaths within 24 h of effective resuscitation (*n* = 6). However, note that the survival rate, ranging around 58% in the CA group, increased over time to approach 80% during the last series of experiments, mainly due to a significant progressive improvement in our technical skills in manipulating such young animals. Thus, when mastered this protocol of cardiac arrest followed by resuscitation is reproducible and provide to be a good tool to investigate pathological consequences of CA. The total protocol duration (from curare injection to extubation) was 58.4 ± 16.2 min in the CA group and 33.8 ± 5.5 min in the control group (*p* < 0.001). This significant difference was due to several longer protocol phases for the CA group (resuscitation time and a slower recovery of spontaneous breathing). When comparing the CA and the control group, there were no statistically significant differences in weight, sex, and age (Table 1). In the CA group, the mean duration of bradycardia (HR < 120/min) was 420.8 ± 49.4 s. Prior to resuscitation, most animals showed a pattern of extreme bradycardia (HR < 60/min) or asystole; no expression of ventricular tachycardia or ventricular fibrillation was recorded. In the control group, HR remained stable throughout the procedure (370 ± 40/min).

**Table 1:** Characteristics of animals included in the study.

**Characteristic**	**CA (*n* = 28)**	**Control (*n* = 10)**	**Stats**	***p***
**Characteristics by group**				
Mean weight (gm)	37.37 ± 6.41	35.20 ± 4.66	*t* = 0.98	0.34
Sex ratio (M/F)	3.50	1.00	χ^2^ = 2.70	0.10
Age (PND)	14.96 ± 1.11	14.60 ± 0.52	U = 125.00	0.61

### MRI Analysis

Following the CA procedure, MRI observations were conducted on these animals at two distinct times: 1 day following the CA to investigate potential acute effects of CA, and 7–10 days later to evaluate more slowly developing cerebral damages. We focused on brain structures known to be affected by HIE in humans, such as the hippocampus, striatum and thalamus. In the CA group compared to the control group, we did not observe any massive anatomical lesions in conventional T2-weighted imaging (data not shown). Furthermore, no significant differences between the groups regarding mean ADC values in the three structures studied were evident (Table 2), whatever the period post-CA. These results thus indicated that no major anatomical anomalies were detectable in CA-subjected animals using MRI. However, it remains possible that less pronounced anatomical deficits might exist but could not be revealed by MRI, especially due to the small size of the animals used in the present study.

**Table 2:** ADC values at day 1 and days 7–10 after experimental procedure.

**Structure (Day post-procedure)**	**CA**	**Control**	**Stats (U)**	***p***
**Mean ADC value (10**^**−4**^ **mm**^**2**^**/s) by group**				
Hippocampus (D1)	7.73 ± 0.95	8.36 ± 0.82	6.00	0.79
Striatum (D1)	8.17 ± 0.43	7.49 ± 0.57	1.00	0.67
Thalamus (D1)	7.86 ± 0.56	7.99 ± 0.72	4.00	1.00
Hippocampus (D7–10)	8.48 ± 1.09	8.65 ± 0.89	4.00	1.00
Striatum (D7–10)	8.66 ± 0.93	8.02 ± 1.32	3.00	0.80
Thalamus (D7–10)	8.40 ± 1.09	7.97 ± 1.01	3.00	0.80

*ADC, Apparent Diffusion Coefficient; D1, day 1; CA, Cardiac Arrest*.

### Histological Analysis

**Figure 2:**
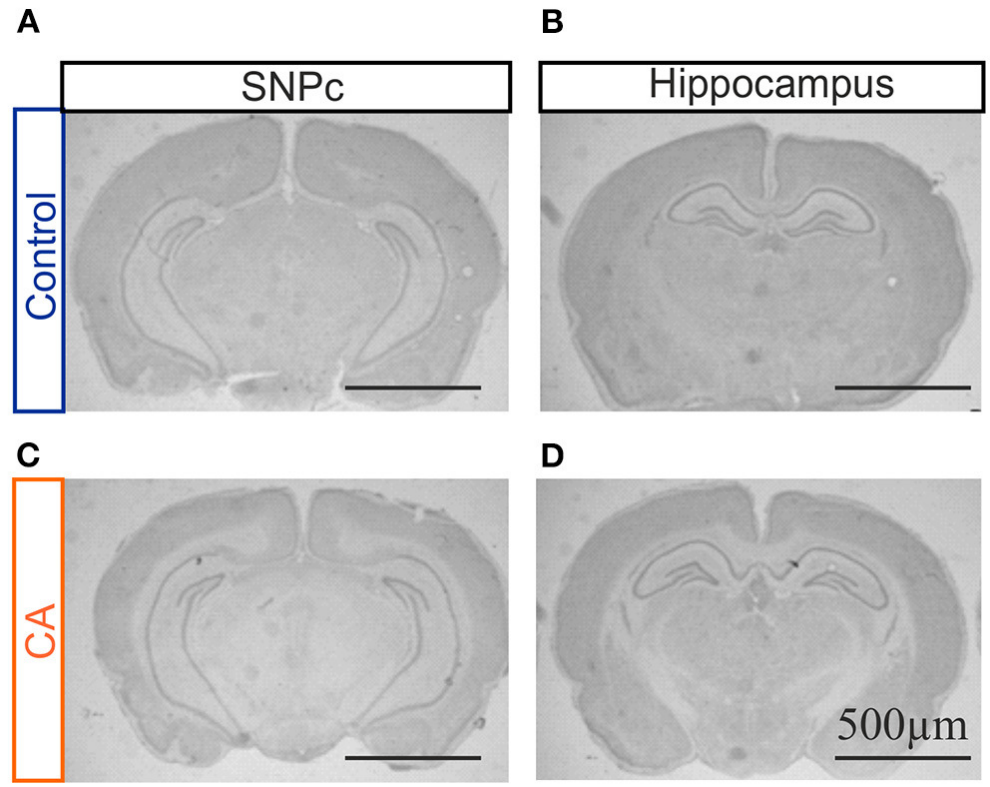
Photographs of coronal brain slices obtained from a control rat **(A**,**B)** and a rat previously subjected to a cardiac arrest (CA) **(C**,**D)** at the level of the substantia nigra pars compacta (SNPc; **A**,**C**) and the hippocampal region **(B**,**D)**. Slices were stained with cresyl violet. Note the absence of any major anatomical damage in the CA brain.

In order to complement our MRI assessment and to proceed to a finer characterization of potential anatomical lesions in brain tissue, we conducted a qualitative histological analysis with Cresyl Violet staining on brain slices obtained from animals having been subjected to the CA paradigm and compared these to the equivalent tissue regions from control animals. A careful observation was made of all brain structures, but here again with a specific interest in the hippocampus, striatum and thalamus. However, when compared to control brains, slices from CA animals showed a typical anatomy, as illustrated for the hippocampus in Figure 2. Similar results were obtained for tissue harvested 1 day after the CA and at 7–10 post-trauma, indicating (as from MRI) that significant brain lesions resulting from a CA, if any, do not emerge in the 10 day time period following the injury or are very spatially limited.

**Figure 3:**
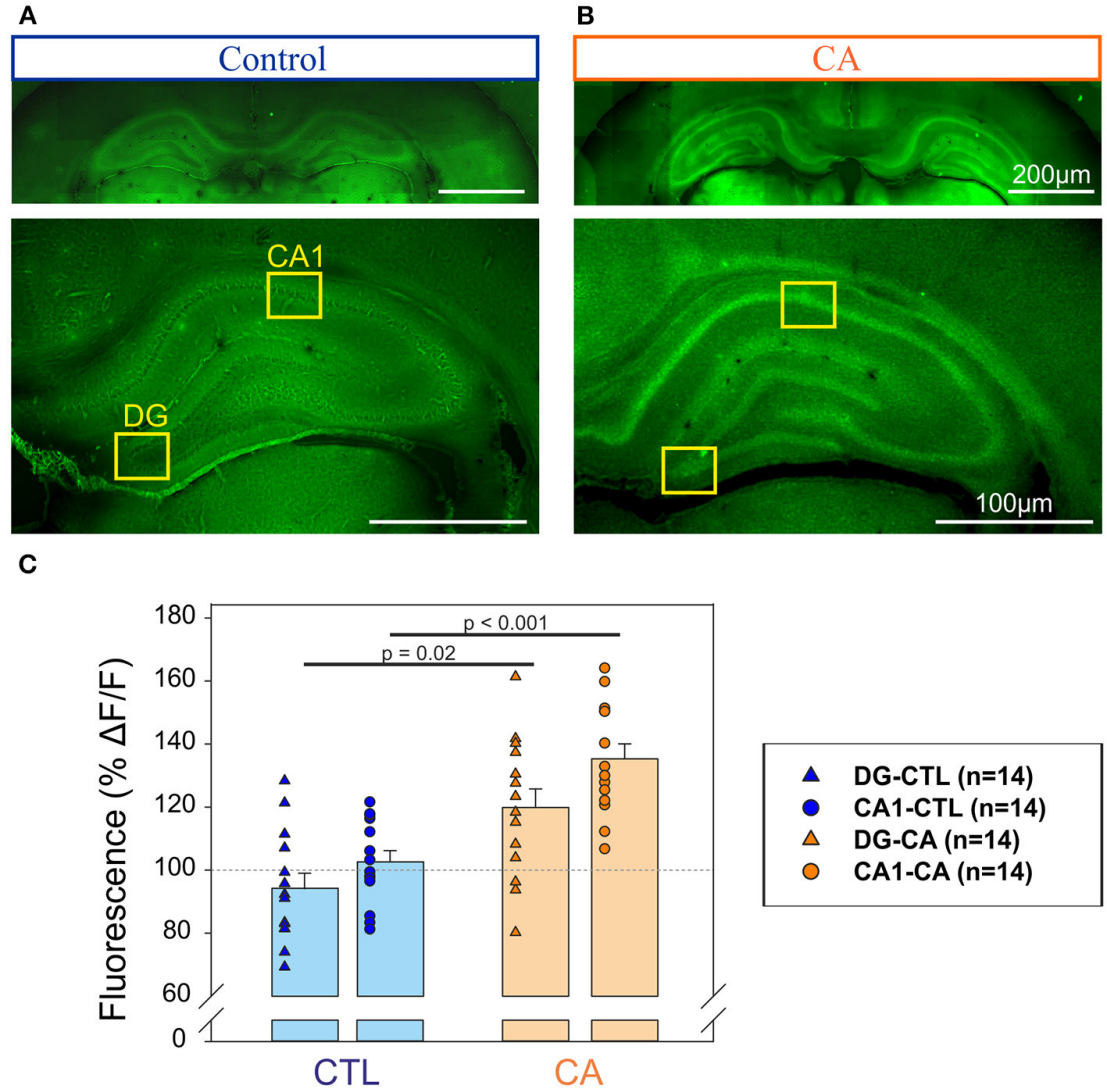
Detection of neurodegeneration in the hippocampus with Fluoro-Jade. Photographs of coronal slices obtained for control animals **(A)** and animals exposed to a CA at Day 1 **(B)** at two different magnifications (top, bottom). Note that in each case top and bottom images have been obtained from different slices. The yellow rectangles delineate the Dentate gyrus (DG) and the CA1 region of the hippocampus in which fluorescent measurements have been performed to be compared to background signal. **(C)** Histograms representing the % of relative change of fluorescence (ΔF/F) in the regions of interest (DG, triangles and CA1, circles) for control (*n* = 14, blue symbols) and CA1 (*n* = 14, orange symbols). A significantly stronger intensity of fluorescence in the DG and CA1 regions of the hippocampus in the CA tissue, suggesting a higher degree of neuronal degeneration.

In a second series of experiments, we sought signs of neuronal degeneration in brain tissue following the acute CA procedure. To this end, Fluoro-Jade B staining was used to identify such a process. A greater Fluoro-Jade B staining in the Dentate gyrus (DG) and the CA1 region of the hippocampus was found in rats from the CA group compared to these same brain regions in the control group when brain sampling was performed at day 1 after the CA procedure (*n* = 14 measurements from 7 slices obtained from 3/4 different animals in each group; Figure 3). We found an intensity of fluorescence that was 35 ± 4.7% significantly (*p* < 0.001) higher in the CA1 of the CA group compare to control slices, and 19 ± 5.9 % significantly (*p* = 0.02) higher in the DG in CA group compare to control slices (Figure 3C). In contrast, inspection of other brain areas did not reveal any signs of abnormal neuronal death. Thus, consistent with other results of our study (see below), our anatomical investigation of brain tissue failed to reveal any widespread, evident anatomical defaults in rats having previously suffered from a 9 min CA, although there were early signs of region-specific neurodegeneration, localized to the hippocampus.

### Behavioral Analysis

In a further exploratory approach, we performed a series of behavioral measurements to test for functional correlates of this hippocampal neurodegeneration. The main parameters evaluated were motor function, anxiety and a form of hippocampal-dependent memory. These tests were conducted on adult male rats (PND 56–80) in order to assess for stable and long-lasting deficits resulting from the 9 min cardiac arrest applied previously at PND 14–16.

#### Open Field

**Figure 4:**
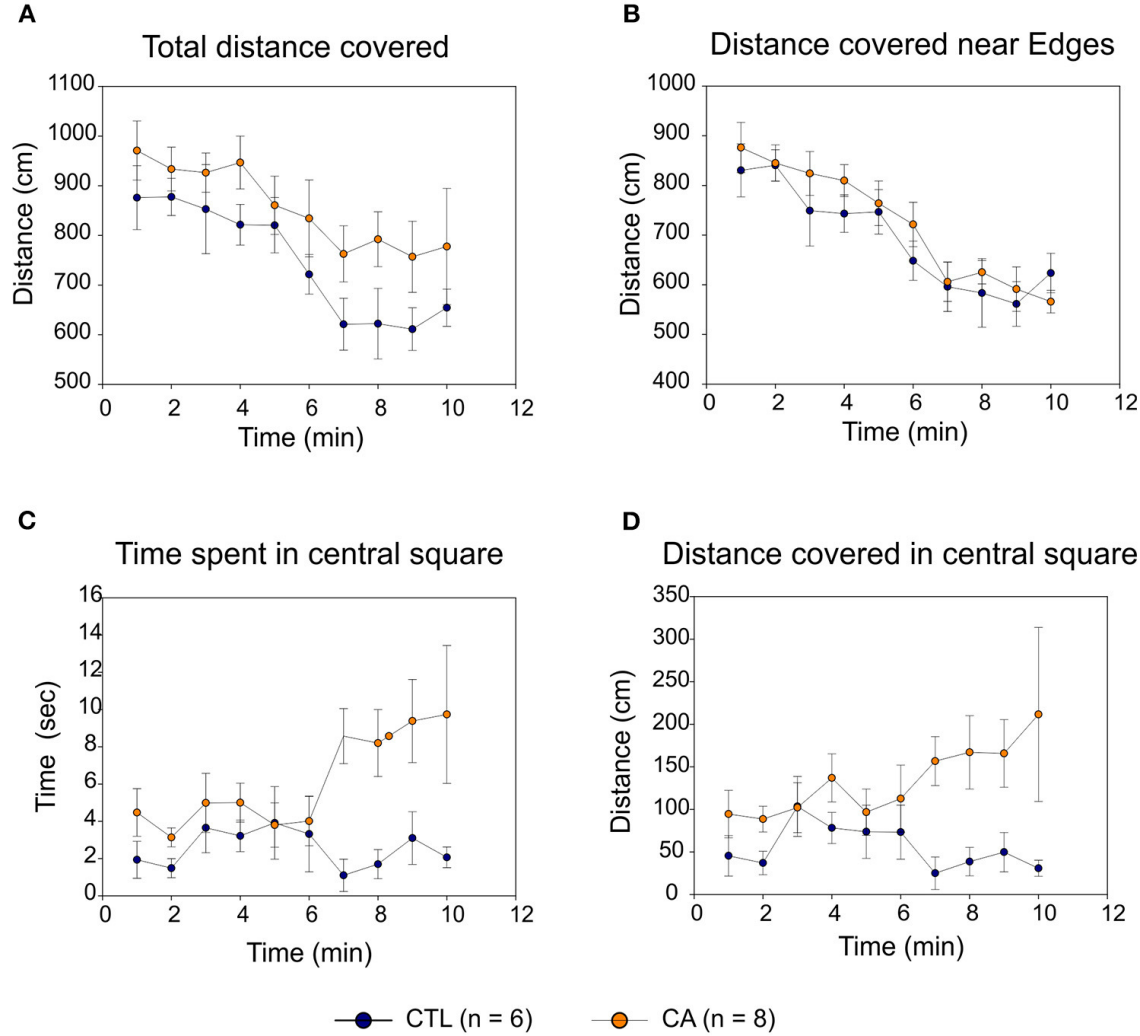
Open Field behavioral testing. The graphs indicate distances covered in the open field over time **(A)**; distances covered near the field perimeters **(B)**; times spent in the central square **(C)** and distances covered in the central square **(D)** for the control group (CTL; blue symbols) and the cardiac arrest (CA) group (orange symbols). Values are expressed as Mean ± SE.

In both CA and control groups, locomotor activity in the open field gradually decreased over time with similar trajectories, with both the total distance covered (Figure 4A) and the distance covered near the area edges (Figure 4B) decreasing significantly (*F*_(9.108)_ = 6.24, *p* < 0.01 and *F*_(9.108)_ = 14.18, *p* < 0.01, respectively). This suggests the development of an habituation phenomenon that was apparently similar in the two experimental groups (absence of Time^*^Group effect: *F*_(9.108)_ = 0.29, *p* = 0.98). Consequently, locomotor activity was comparable in both groups: there were no significant inter-group difference either in total spontaneous displacement (*F*_(1.12)_ = 4.05, *p*=0.07) or in displacement close to perimeter edges (*F*_(1.12)_ = 0.99, *p* = 0.34).

In contrast, rats in the CA group spent significantly more time in the central square (Figure 4C) than control group animals (*F*_(1.12)_ = 13.49, *p* < 0.01), and they were more likely to explore the central square as indicated by the consistently higher distances that they traveled whilst in this more exposed area (*F*_(1.12)_ = 6.51, *p* = 0.03; Figure 4D). It is noteworthy that although this latter difference was not sufficiently elevated to convey mean values for total distance covered (Figure 4A) into a range that was significantly different from control (*p* = 0.07), it very likely participated in the trend observed. Furthermore, for all of these parameters, the “Time” variable had no significant effect. There is also no statistically significant Time^*^Group interaction, although there was an evident trend toward the development of such a group effect in both the amount of time spent, and the distance traveled, within the central square (*F*_(9.108)_ = 1.79, *p* = 0.08). Taken together, therefore, these results strongly suggest that rats in the CA group exhibited a level of behavioral anxiety that was lower than that of their non-CA counterparts.

#### ECM Analysis

**Figure 5:**
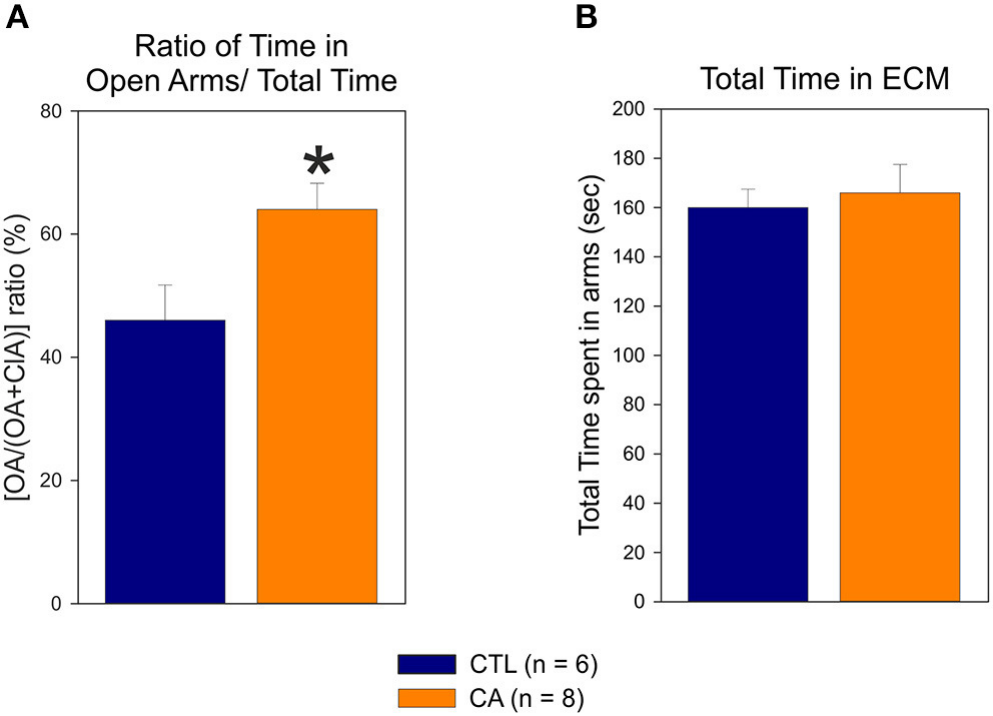
Elevated Cross Maze test: the histograms represent the ratio of time spent in the open arms (OA) relative the total time spent in the maze **(A)** and the total time spent in both open and closed arms (ClA) **(B)**. Values are means ± SE obtained from 6 control (CTL, blue bar) and 8 cardiac arrest (CA, orange bar) animals. Asterisk indicates significant difference (*p* < 0.05, Mann Whitney test).

In the elevated cross maze test, the proportion of time that rats spent in the open arms (i.e., the [OA/(OA+ClA)] ratio) was significantly higher in the CA group than in the control group (64 ± 12 vs. 46 ± 14%, U = 6.00, *p* = 0.02) (Figure 5A). No significant difference was observed between the CA and control groups regarding the total time spent in both open and closed arms of the maze (166 ± 32.4 vs. 160 ± 18.2 s, U = 18.00, *p* = 0.49; Figure 5B). This indicates that rats in the CA group are more likely to explore open arms than rats in the control group, although this is not due to greater locomotor activity. These findings are therefore consistent with those obtained from the Open Field analysis described above, further supporting the conclusion that a CA leads to a decrease in physiologic unconditioned fear responses.

#### Fear Conditioning Test

**Figure 6:**
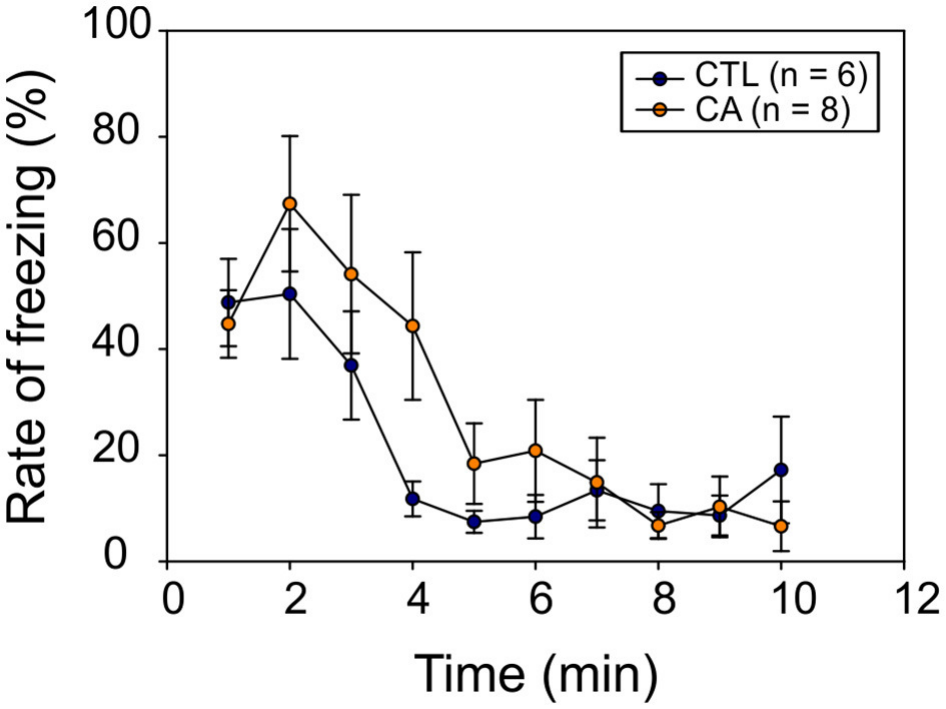
Recall phase of the fear conditioning test. The graph represents the mean behavioral freezing rate (in %) over time obtained for 6 control (CTL, blue symbols) and 8 CA animals (orange symbols). Data are expressed as Mean ± SE.

In a final set of behavioral experiments, we investigated potential memory impairment resulting from a 9 min CA. For this, we used a classical fear conditioning test involving the behavioral freezing response (see section Materials and Methods). During the conditioning phase, the freezing rate increased significantly over time (*F*_(7.84)_ = 73.03, *p* < 0.01), but without any significant difference between groups (*F*_(1.12)_ = 0.04, *p* = 0.84), nor was there a significant Time^*^Group effect (*F*_(7.84)_ = 0.77, *p* = 0.62) observed. Thus, animals in both groups appeared to gradually develop a conditioned fear response in a comparable way. Subsequently, during the recall phase, we observed a significant decrease in the freezing rate over time (*F*_(9,108)_ = 12.86, *p* < 0.01), and again, there was no significant difference between the CA and control groups (*F*_(1,12)_ = 0.90, *p* = 0.36) (Figure 6). There was also no interaction between time and group variables (*F*_(9.108)_ = 1.53, *p* = 0.15). Thus, altogether these results do not support the possibility of a long-term memory impairment in animals subjected to a CA.

## Discussion

In this study, we have successfully developed a low-invasive protocol for an acute (9 min) hypoxic cardiorespiratory arrest in PND 14–16 rats. We demonstrated that this protocol leads specifically to a neurodegeneration in the CA1 region of the hippocampus at day 1 after CA, an effect that is associated with the later expression of anxiolytic-like behavior at adult stages but without any loss in memory capabilities. However, the use of MRI for gross anatomy assessment and cresyl violet staining for finer observations failed to reveal any significant anatomical defects that could underlie this behavioral change. Nevertheless, our data indicated that a short-lasting cardiac arrest triggers abnormal neuronal degeneration, at least in the hippocampus, that subsequently becomes discernible a few days later. Thus, our model could be useful to better understand the neurophysiological consequences of an HIE occurring at young ages.

### Behavioral Changes Induced by HIE

Rats that had undergone our experimental CA procedure were found to develop reduced levels of unconditioned fear responses (anxiolytic-like behavior) in adulthood, as evidenced by both Open Field and ECM assessment. Similar results were obtained by Wang et al. following an intracerebral injection of LPS in PND 5 rats (27), where atrophy and neuronal death in the CA1 region leading to adult memory deficits were not only observed, but also less anxiety-like (anxiolytic-like) responses in the ECM task. This could be explained by lesions to the ventral hippocampus, since this structure also plays a role in the regulation of emotional behavior (28). Indeed, lesions of the septo-hippocampal system, which is the neural substrate of behavioral inhibition, may lead to a decrease in fear and anxiety reactions (29, 30). However, the consequences of hippocampal lesions for anxiety and fear behaviors remains unclear since other studies have reported the expression of hyper-anxious behavior after prenatal hypoxia-ischemia (31) or after neonatal hippocampal lesions in non-human primates (32). Therefore, additional experiments are required to unequivocally associate defaults in the hippocampus with this anxiolytic-like behavior in the context of our paradigm used to induce HIE. Notably, we could use tests that would allow us to discriminate this anxiolytic-like behavior from depression, but the latter is probably not importantly developed here as the general locomotor behavior is normal and not reduced as expected in the case of depression. In addition, it could be relevant to measure plasma corticosterone levels to characterize neuroendocrine stress responses during a stressful procedure, in order to quantitatively assess the anxiolytic-like behavior shown by CA rats.

Furthermore, in our case, we did not find any significant memory deficits in rats of the CA group, although this conclusion thus far derives from a single test (Fear conditioning test) and should be complemented with experiments using other memory tests, such as with Morris water maze procedures. Interestingly, it has been shown that a selective lesion of the ventral hippocampus in rats is able to induce a decrease in unconditioned fear responses, as well as decreased neuroendocrine stress responses, but without impairing contextual fear conditioning nor spatial navigation (33).

In addition, we did not observe locomotor impairment in rats of the CA experienced animal. However, although a model of severe HIE would be expected to induce significant motor impairment, as observed in neonates (1), this outcome is rarely found even in studies using the Vannucci protocol at PND 7 (34). Accordingly, with our model that evidently produces a mild HIE, associated locomotor deficits might also be relatively moderate and difficult to pin-point without using more specific tests.

Nevertheless, attempting to extrapolate the results from our animal model to the consequences of HIE in humans requires caution when interpreting the severity of a neurological or behavioral deficit. For instance, it is possible that abnormally low unconditioned fear responses, a physiologic defensive behavior, might effectively place a rat at far greater risk than it would in a human. In other words, this anxiolytic-like state in a rat might expose it to a level of vulnerability that in humans is more equivalent to the vulnerability arising, for example, from a hemiparesis that often results from a severe HIE.

### Anatomical Damage Caused by HIE

Following a 9 min hypoxic cardiorespiratory arrest, we expected to detect important anatomical signs of damage to brain tissue. However, MRI inspection performed either in the acute phase (day 1) or after more than a week (days 7–10) failed to reveal any obvious structural alterations, although several methodological factors might be relevant to our employment of this technique. First, the use of isoflurane as an anesthetic could modify cerebral blood flow and then may perturb the consequences of HIE in rats (neuroprotection) (35). Second, the delay chosen between the application of the CA procedure and MRI assessment may not have been the most appropriate. In a first instance, these delays were selected in analogy to the human pathology, but they probably would require adjustment in future investigations. In humans when using MRI, cerebral hypoxic ischemic damage can be visualized by a T1 hyperintense signal appearing between 3 and 5 days following a T2 hypointense signal occurring between postnatal days 6 and 10. Later, depending on the severity of the lesion, atrophy of the basal ganglia and thalamus, persistent cortical T2 hypersignals, thinning of the corpus collosum, and sometimes cavitation or even multi cystic encephalopathy can be observed (5). During the first 2–3 days of life, however, these structures can appear normal under MRI. However, DWI sequences are able to detect lesions in the acute phase (36) that are closely correlated with histopathological data (37), but with two limitations: an underestimation of the lesions if performed too early (<24 h) and a sensitivity that declines after 7 days (5). Third, T1-weighted imaging could also have been performed in addition to T2 imaging, since subacute injuries (before 5 days after a CA in the human newborn) are not visible in the latter sequences. Finally, we cannot rule out the possibility that investigating brain anatomy of such small rodents using MRI might be confronted with an insufficient spatial resolution that prevents detection of fine tissue damage.

To try to circumvent this potential problem we complemented our first set of MRI observations with a series of experiments using standard cresyl violet histological staining of thin brain slices. Here again, however, no obvious signs of anatomical lesions in different brain structures could be detected following our HIE protocol. One possibility is that either our CA procedure was not sufficiently disruptive to induce significant tissue lesions, or that once again the post-CA timing of our anatomical observations was inappropriate: making observations after only 1 day might have been too premature for processes of tissue necrosis to have occurred or be sufficiently advanced to become detectable. Thus, in the case of such limited alterations, a densitometric analysis and/or a more precise neuronal cell count could have provided a finer anatomical assessment that revealed milder tissue lesions. In any case, if our model actually induces brain lesions, they are mild at most. Usually, tissue resistance to hypoxia varies across species (38). Therefore, it is possible that rats have a better cerebral tolerance to hypoxia than humans (39), which could explain our incapability to reveal severe lesions after a protocol of profound hypoxia such as ours.

Nevertheless, our results do suggest that our model of HIE induces hippocampal damage. Specifically, Fluoro-Jade labeling of neurons in the CA1 region of this structure, indicating the occurrence of abnormal neurodegenerative processes (20). Importantly, such a specific structural loss could underlie the behavioral changes observed in our animals after brain oxygen deprivation (see above).

### Validity of Our Model and Perspectives of Improvement

Our model differs in several ways from that developed by Fink et al. (14). First, in our study we used younger rats, PND 14–16. Alternatively, the ideal age for an HIE model is in fact at younger PND 7–13, since cerebral development in this stage range is comparable to that of a full-term newborn in terms of myelogenesis and the expression oligodendrocyte maturation markers (15, 40). In a preliminary set of experiments, we attempted to use such younger rats but were confronted with a high degree of mortality, mainly due to the difficulty of intubating animals at these ages. Thus, considering our aim to mimic human neonatal hypoxic CA and resuscitation as closely as possible from a physiopathological perspective, we used the youngest possible rats; although we are aware that this age range is not ideal for a neonatal HIE model. Nevertheless, during the course of our study the survival rate increased over time, reflecting the improvement of our protocol and of our intubation technical skills. Applying our experimental procedure to younger PND 12–13 rats to continue developing and validating our animal model for HIE is therefore conceivable for future experiments.

Furthermore, compared to the Fink model we chose a longer hypoxia duration (9 min instead of 8 in the Fink model) in order to increase the probability of neurological lesions. Our preliminary investigations with a 10 min CA duration resulted in a high mortality rate. It therefore seems impractical to further increase hypoxia duration with our protocol, although the problem could be alleviated by modifying some parameters of our experimental procedure, such as by applying moderate hypoxia before switching off the ventilator.

In addition, our model is much less invasive than Fink's model, which could constitute a strength as well as a weakness. On one hand, by avoiding surgery, we reduce the duration of the protocol, the duration of exposure to isoflurane, risk of infection, and blood spoliation. On the other hand, however, without catheterization, resuscitation must be performed “blindly”: our ventilatory parameters are set up arbitrarily since we cannot perform arterial blood gas analysis; and without invasive blood pressure measures we only have an indirect indication of the occurrence of a cardiac arrest, which is unreliable because terminal rhythms such as electromechanical dissociation are common.

The relatively high mortality rate observed after our CA procedure can appear as a limitation. However, we emphasize that this rate has been calculated over the entire project (including some initial phases devoted to establish steps of our paradigm) and drastically decreased over the course of the study, reflecting the improvement of our technical expertise. Indeed, for example, in order to limit animal death we tested several epinephrine administrations routes, the retro-orbital route appearing to be the best. Thus, the residual mortality observed during the last weeks of experimentation reaching <20% is, in our opinion, acceptable and remains consistent with that expected for such experiments.

## Conclusion

Addressing HIE, translational research faces a challenge: there are few animal models that reproduce a pathophysiology similar to that found in the human newborn. Our aim was to develop a model approaching this as closely as possible, despite interspecies differences in cardio-vascular and cerebral tolerance to hypoxia.

We successfully developed a low-invasive protocol for an acute (9 min) hypoxic cardiorespiratory arrest in PND 14–16 rats. Since no evidence was found for memory or locomotor impairments after CA, nor evident anatomical lesions, our rat model does not represent a severe case of HIE. However, our data does point to a persistent hippocampal and likely associated behavioral impairment in CA-exposed animals, and consequently, our study should be considered as a preliminary step in the development of a new animal model for a mild form of HIE. Further in-depth investigation of the molecular, anatomical and behavioral consequences of our ischemic protocol is now required, and a refinement of the CA protocol itself should also be considered to more accurately replicate the neurobiological processes occurring in young humans.

## Data Availability Statement

The raw data supporting the conclusions of this article will be made available by the authors, without undue reservation.

## Ethics Statement

The animal study was reviewed and approved by Ethics Committee of the University of Bordeaux.

## Author Contributions

OB: study conception. OB, MT-B, and JG: study design. OB and MT-B: coordination. JG, OB, LC, and MT-B: realization of the experimental procedure. JG, OB, and MT-B: data interpretation and drafting manuscript. All authors agree to be accountable for the content of this work.

## Conflict of Interest

The authors declare that the research was conducted in the absence of any commercial or financial relationships that could be construed as a potential conflict of interest.
